# Regenerative Medicine in Diabetes

**DOI:** 10.1016/j.mayocp.2015.01.019

**Published:** 2015-04

**Authors:** Aleksey Matveyenko, Adrian Vella

**Affiliations:** 1 Division of Physiology and Biomedical Engineering, Mayo Clinic, Rochester, MN; 2 Division of Endocrinology & Metabolism, Mayo Clinic, Rochester, MN

**Keywords:** Type 1 diabetes, stem cells, β-cell development, insulin secretion

## Abstract

Diabetes is a common, multisystem disease that results in hyperglycemia due to a relative or absolute insulin deficiency. Improved glycemic control decreases the risk of development and progression of microvascular and, to a lesser extent, macrovascular complications as well as preventing symptomatic hyperglycemia. However, complex treatment regimens aimed at improving glycemic control are associated with an increased incidence of hypoglycemia. On paper at least, cellular therapies arising from reprogramed stem cells or other somatic cell types would provide ideal therapy for diabetes and the prevention of its complications. This has led to intensive efforts at growing β-cells from various sources. In this review, we provide an overview of β-cell development as well as the efforts reported to date in terms of cellular therapy for diabetes. Engineering β-cell replacement therapy requires an understanding of how β-cells respond to other metabolites such as amino acids, free fatty acids and ketones. Indeed, efforts to date have been characterized by an inability of cellular replacement products to adequately respond to metabolites which normally couple the metabolic state to β-cell function and insulin secretion. Efforts to date intended to capitalize on current knowledge of islet development and stimulus-secretion coupling of the β-cell are encouraging but as yet of little clinical relevance.

## General Introduction

Diabetes is a common, multisystem disease that results in hyperglycemia due to a relative or absolute insulin deficiency and arises from a complex interaction between genes and the environment ^1^. The presence or absence of disease is defined by hyperglycemia, the degree and duration of which leads to microvascular complications such as retinopathy, nephropathy and neuropathy. Absolute insulin deficiency is typically encountered in immune mediated or type 1 diabetes (T1DM) where an immune response results in destruction of β-cells – the site of endogenous insulin production. In contrast, in type 2 diabetes (T2DM) insulin deficiency, while, due in part to loss of functional, responsive β-cells, is not absolute but relative to the impaired insulin signalling present in this disorder ^2^.

Improved glycemic control decreases the risk of development and progression of said complications as well as preventing symptomatic hyperglycemia. However, complex treatment regimens aimed at improving glycemic control are associated with an increased incidence of hypoglycemia. This is one of the more feared complications of diabetes treatment and can lead to significant neurocognitive dysfunction and impairment of quality of life. This has led to a quest for alternative therapeutic strategies. On paper at least, cellular therapies arising from reprogramed stem cells or other somatic cell types would provide ideal therapy for diabetes and its complications.

## Clinical Need

T1DM, and to a lesser extent, long-standing T2DM require multiple daily injections of insulin to achieve good glycemic control. This requires considerable commitment and resilience on the part of the patient. Indeed achieving glycemic targets while avoiding hypoglycemia may be difficult for some. The more feared complication associated with longstanding T1DM is hypoglycemia unawareness when a defective counter-regulatory response to hypoglycemia results in frequent hypoglycemia with little prodromal symptoms. As a consequence, affected patients are at risk of cognitive dysfunction and hypoglycemic seizures and are often unable to work or drive ^3,4^.

To date treatment has consisted of whole-organ pancreas transplantation or islet transplantation which can restore endogenous insulin secretion and improve microvascular complications. However, transplantation exposes patients to both surgical complications and the consequences of immune suppression including opportunistic infection and the toxicities of immunosuppressants ^5^.

In addition to the problem of achieving and maintaining glycemic control, the microvascular complications of diabetes most especially neuropathy and its consequences may lend themselves to cell replacement therapies. Diabetic neuropathy is a common cause of foot ulceration, deformity and amputation. Autonomic dysfunction especially affecting the gastrointestinal tract causes disruption to daily activities and may further complicate glycemic control as well as increasing mortality ^6^. It is in this context that better therapies for diabetes and its complications are needed.

## The Embryonic Development of β-cells

The pancreas arises from dorsal and ventral epithelial buds from the endoderm of the posterior foregut on approximately the 10^th^ embryonic day (the timing is specific to rodents). The epithelial cells evaginate into surrounding mesenchyme forming an accretion of multipotent progenitor cells (MPCs) surrounding a central lumen. All subsequent progenitor cells as well as the different cell types present in the adult pancreas (i.e. acinar, ductal and islet cells) arise from these accretions ^7^. By the 13^th^ day of embryonic development, the dorsal and ventral pancreatic buds have fused and are accompanied by expansion of the MPCs with cells exhibiting apicobasal polarity and microlumen formation. The number of MPCs allocated to the initial pancreatic buds seems to control ultimate organ size, suggesting that the MPCs giving rise to the adult pancreas are already somewhat pre-differentiated and are limited in the number of cell cycles they are able to undergo while expanding tissue, developing organ architecture and differentiating into defined cell lineages ^8^. The first wave of endocrine cell differentiation is predominantly α-cell differentiation although some cells express Ghrelin (ε-cells), Pancreatic Polypeptide and Somatostatin (δ-cells). Subsequent development leads to delamination and budding of endocrine cells into architecture reminiscent of the adult pancreas. There has been a suggestion that a greater proportion of adult β-cells arise from the dorsal as opposed to the ventral bud implying that the milieu present within the dorsal bud may be more apposite to the differentiation of MPCs into functioning β-cells ^9^.

What drives the development of endocrine cells in the embryonic pancreas? It is apparent that a variety of factors interplay at various stages of development including factors that are extrinsic to MPCs and arise in the adjacent mesenchyme. There are also signals that are intrinsic to the developing epithelium acting in a paracrine fashion. The timing and combination of these signals activates a series of gene regulatory networks through a variety of transcription factors that have context-specific roles in specifying cell fate. *Ngn3* is a basic Helix-Loop-Helix (bHLH) transcription factor that is identified early in the development of the dorsal pancreatic bud. Mice lacking *Ngn3* fail to develop pancreatic endocrine cells and die from postnatal diabetes. *Ngn3^−/−^* mice lack expression of Isl1, Pax4, Pax6, and NeuroD implying that Ngn3 is an upstream regulator of these transcription factors ^10^.

The homeobox transcription factor Pdx1 is required for transcription of multiple β-cell genes, including those for insulin and glucokinase (an enzyme that phosphorylates glucose) and for developmental formation of the entire pancreas. Mice homozygous for a null mutation in *Pdx1* fail to develop a pancreas, whereas restricted inactivation of Pdx1 in the murine β-cell produces insulin deficiency and diabetes. Pancreatic agenesis has also been reported in human subjects homozygous for a loss-of-function *PDX1* mutation, and subjects heterozygous for *PDX1* develop a form of maturity-onset diabetes of the young (MODY) ^11^. The genetic mutations associated with MODY to a greater or lesser extent provide insights into the development of β-cells. Binding sites for the MODY genes *PDX1*, *HNF1A*, and *HNF4A* have been identified in the *PAX4* promoter, suggesting that MODY genes may be upstream regulators of genes critical for islet cell formation and islet function in the pancreas ^12^. Mutations in *NEUROD1* produce a form of MODY in humans characterized by defective insulin secretion ^13^. Mice lacking NeuroD1 have marked reductions in islet cells with arrested β-cell development.

## Characteristics of Mature β-Cells – Insulin Secretion

**Figure 1:**
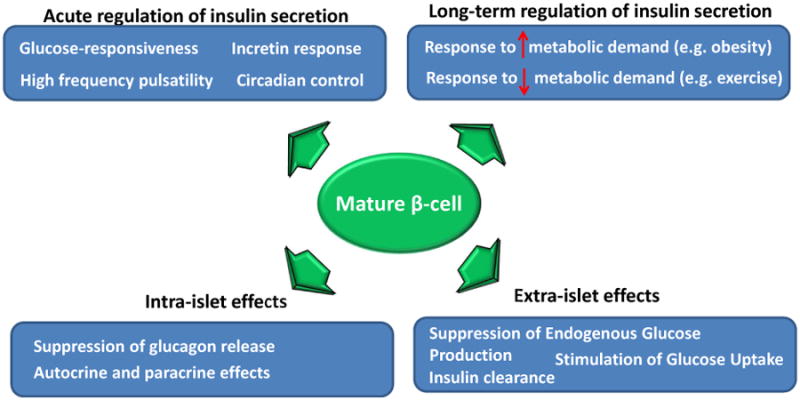
Functional characteristics of beta-cells essential for proper glucose control *in vivo*

In health glucose concentrations during fasting are tightly regulated within a relatively narrow band. During periods of fasting, glucose in the bloodstream originates from the liver which is under tonic inhibition by insulin. Glucose itself stimulates its peripheral uptake and can suppress its own production. Counter-regulatory hormones such as catecholamines and glucagon can raise glucose concentrations if necessary. During fasting, insulin secretion occurs in a pulsatile fashion with pulses increasing in amplitude and frequency when glucose concentrations rise ^2^ (Fig. 1). Indeed β-cells also respond to other metabolites such as amino acids, free fatty acids and ketones. In response to meal ingestion, there is an exponential rise in insulin secretion so that the postprandial increment in glucose concentrations (and other aforementioned metabolites) are contained and return to near-fasting concentrations within two hours. β-cell function is essential to metabolite homeostasis and is mediated by its ability to respond to metabolites – sometimes termed metabolite coupling factors because they are products of intermediary metabolism that couple the metabolic state to β-cell function – by secreting insulin ^14^.

**Figure 2:**
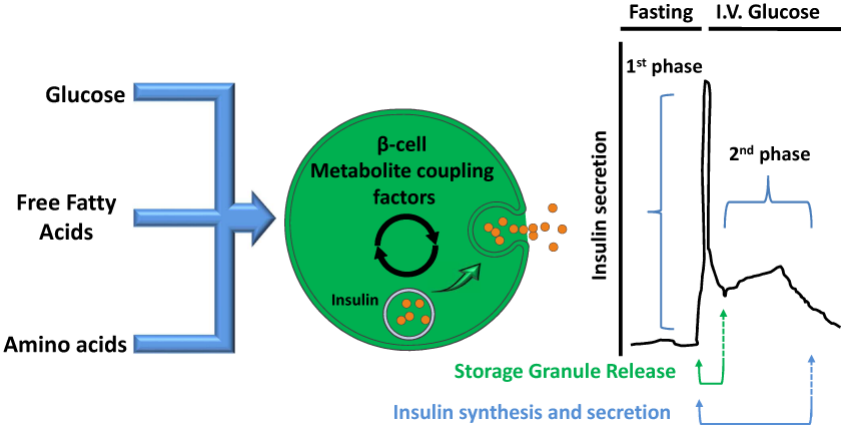
Metabolites provoking insulin secretion *in vivo* and the resulting phases of insulin secretion

Isolated β-cells, perfused pancreata, rodent and other animal models as well as humans exhibit a biphasic response to intravenous glucose – the first phase is rapid but not sustained and is thought to represent release of preformed secretory granules in response to the rapid rise in glucose concentrations (Fig. 2). The second phase likely represents insulin synthesis and secretion in response to hyperglycemia. Although these two phases of insulin secretion are not apparent in response to oral challenges in humans, mathematical modelling can nevertheless differentiate the static (synthesis and secretion) from the dynamic (release of preformed granules) components of insulin secretion. Both components are necessary for glucose homeostasis although disruption in the static phase of insulin secretion is more commonly observed in diabetes. Pharmacological agents such as glucagon-like peptide-1 receptor agonists preferentially increase static insulin secretion ^15^.

Genome-Wide Association Studies examining the predisposition to T2DM have identified several genes which affect β-cell function – some of which are important for insulin granule assembly - while others regulate stimulus – secretion coupling ^1^. Glucose metabolism in β-cells is unique in that it is governed by substrate availability. This is made possible by the rapid equalization of intracellular glucose with extracellular glucose by the Glut-1 transporter in humans. In addition, glycolysis and consequent carbon entry into the TCA cycle which is the primary fate of glucose in the β-cell is regulated by glucokinase, a hexokinase with low glucose affinity. Indeed, half-maximal activity is achieved at a glucose concentration of ∼8mMol ^14^. Other hexokinases which are more active at lower concentrations of glucose are repressed during maturation to functional β-cells ^16^. The rate of glucose phosphorylation ultimately determines the rate of ATP production which controls membrane potential and insulin release through the tandem operation of the nucleotide sensitive K_ATP_/SUR1 channel complex and the voltage-sensitive L-type Ca^2+^ channel mediating insulin granule exocytosis ^14^.

The ultimate goal of regenerating β-cells would be – at least at a functional level – to recapitulate the glucose-responsiveness of mature islets coupled with the ability to synthesize and secrete bioactive insulin in amounts that can achieve glucose homeostasis. Hypoglycemic disorders, whether genetic or acquired, result in inappropriate insulin secretion for the prevailing glucose concentrations and illustrate the pitfalls of dysregulated insulin secretion. On the other hand the islets of patients with long-standing T2DM, while still able to secrete insulin exhibit a delayed and decreased response to various secretagogues and oral challenges. Cell replacement therapy for T1DM will have to thread a path between the Scylla of inappropriate insulin secretion and the Charybdis of defective metabolite responsiveness.

## Preclinical studies–cell replacement approaches in diabetes overview)

**Figure 3:**
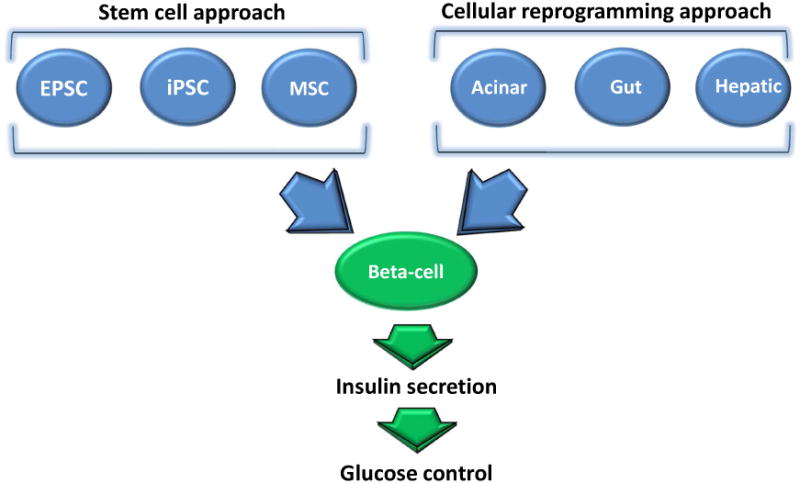
Methodological approaches toward beta-cell replacement in diabetes

Absolute or relative loss of pancreatic β-cells (β-cell mass) is a key pathophysiological event precipitating development of hyperglycemia in both type 1 (T1DM) and type 2 (T2DM) diabetes mellitus. Whereas in T1DM β-cell loss occurs as a result of an autoimmune attack leading to near total loss of β-cell mass, in T2DM β-cell loss is more gradual and manifests as a reduction of β-cell mass ranging from 50 to 65% compared to non-diabetic controls ^17^. Given the critical importance to maintain appropriate β-cell mass for glycemic control ^18^ and consequent prevention of chronic complications, it has become imperative to develop novel therapeutic strategies to replenish the deficit in β-cell mass in diabetes. In the past few decades a number of regenerative approaches have emerged. These approaches include but not limited to β-cell replacement therapy from: 1) embryonic pluripotent stem cells (EPSCs), 2) induced pluripotent stem cells (iPSC) 3) Mesenchymal adult stem cells (MSC), 3) reprogramming/transdifferentiation of various non-β cell types (e.g. acinar cells and entero-endocrine cells) and 4) induction of replication of existing β-cells. A number of recent comprehensive reviews have focused on intracellular signaling mechanisms driving induction of β-cell proliferation ^19,20^, consequently the focus of this discussion will be on examining recent advances in stem cell based and cellular reprogramming approaches (Fig. 3).

## Preclinical studies -EPSC, iPSC and MSC utility as β-cell replacement approach

EPSCs isolated from the inner cell mass of the pre-implantation blastocysts are immortal pluripotent cell lines that display unique capacity to differentiate into somatic cell types of all three germ layers ^21^. Establishment of techniques allowing for isolation and subsequent differentiation of EPSCs into specific cell types has raised tremendous interest and hope that successful differentiation of EPSCs into functional β-cells may provide a therapeutically relevant strategy to replace β-cell loss and ameliorate insulin dependence in diabetes ^22,23^. Protocols seeking successful *in-vitro* differentiation of EPSC's into β-cell lineage typically employ a strategy to recapitulate induction of signaling biological pathways designed to reproduce normal stages of endocrine pancreas development ^24,25^. This is achieved by sequential addition of various growth factors (e.g. Activin A, FGF etc) and signaling pathway activators (e.g. Retinoic acid) employed to modify stem cell fate and differentiation thus directing it towards β-cell fate ^26,27^. Specifically, this process involves recapitulation of key pancreatic formative stages which includes a) formation of definitive endoderm characterized by expression of *SOX 17* and *FOXA2* ^28^, b) induction of pancreatic endoderm characterized by expression of *PDX1* and *HNF6* ^29^, c) endocrine precursor formation highlighted by induction of NGN3 and NEUROD1 ^10^ and eventual progression to d) β-cell lineage marked by expression of Insulin, NKX6.1 and Mafa ^30,31^. Despite continuing protocol refinements β-cell differentiation into mature functional β-cells *in-vitro* remains an area under intense investigation.

A number of studies that reported production of insulin producing cells generated *in-vitro* from human EPSCs noted immature β-cell phenotype which appears to characteristically resemble human fetal β-cells, rather than mature functional adult β-cells ^32,33^. Importantly these cells are often characterized by lack of glucose-stimulated insulin secretion, failed expression of key β-cell transcriptional factors (e.g. NKX 6.1) and abnormal poly-hormonal phenotypes ^32,33^. More recently, two independent groups have published refinements of differentiation protocols utilizing several new small molecules designed to improve β-cell differentiation propensity toward mature β-cell lineage ^30,31^. Specifically, Pagliuca and colleagues ^30^ have reported a refinement to the protocol for generation of β-cell from human EPSCs in-vitro. The new protocol described generation of β-cells that are 1) enriched in β-cell maturation transcription factors (e.g. NKX 6.1), 2) display glucose-responsive insulin release and intracellular calcium elevations in-vitro, 3) possess insulin granules that are structurally similar to primary human β-cells and 4) capable of reducing hyperglycemia once transplanted into diabetic mice ^30^. Interestingly, the protocol also describes a novel method capable of scaling *in-vitro* β-cell production for potential therapeutic application. Although, substantial progress has been made in refining β-cell differentiation protocols from EPSCs; further studies are needed to address the critical question of whether implantation of *in-vitro* generated β-cells will yield a viable therapeutic option capable of maintaining appropriate β-cell function and mass in diabetes.

Some groups have employed an alternative approach to *in-vitro* production of EPSC-generated β-cell. This approach involves *in-vitro* differentiation of EPSC toward PDX-1^+^ pancreatic progenitor stage followed by engraftment/implantation of these pancreatic progenitors into a living organism (typically mice or rats) thus allowing for *in-vivo* maturation process aided by the recipient's microenvironment^34-36^. Indeed a number of studies have utilized this approach and reported potential maturation of EPSC-derived pancreatic progenitors into insulin-producing cells expressing key β-cell maturation factors capable of reversing hyperglycemia once implanted into diabetic mice and rats ^35,36^. Some shortcoming of this approach include a long maturation period reported to range from 2 to 3 months, inconsistent β-cell formation and function, as well as potential propensities for teratoma formation ^26,37^. Nonetheless, the first human trial testing human EPSC potential to replace β-cell loss in T1DM will indeed utilize PDX-1^+^ pancreatic progenitor cell implantation approach with cells seeded into a subcutaneously implanted immuno-solation devices.

In 2007 a seminal paper by Takahashi and colleagues described successful viral reprogramming of human fibroblasts into human induced-PSCs (iPSCs) utilizing four key stem cell transcription factors *OCT 3/4, SOX2, KLF4 and c-MYC* ^38^. This discovery opened the possibility for isogenic cell therapy thus avoiding the complications of immune rejection and also circumventing some ethical issues with harvesting human embryonic stem cells. IPSCs display similar differentiation and proliferative potential to EPSCs and thus have been successfully used to generate islet-like clusters in-vitro from somatic cells capable of glucose-stimulated insulin release and expression of key β-cell maturity factors ^39-42^. Furthermore, iPSC approach has been used to generate disease-specific iPSC and insulin producing cells from patients with diabetes ^39^. Thus iPSCs provide an additional advantage of generating patient-specific cell therapy as well as patient-specific disease modeling and diagnostic potential. On the other hand, questions also remain whether iPSC-derived β-cells obtained from patients with diabetes will retain characteristic molecular abnormalities leading to their eventual failure or reoccurrence of β-cell autoimmunity. The potential for iPSCs-induced β-cell regeneration for prevention of β-cell loss in diabetes remains an area of active investigation ^25^.

Mesenchymal stem cells (MSC) provide another potential avenue for generation of β-cells for insulin replacement in diabetes. MSC is the most ubiquitous adult stem cells that can be harvested from multiple tissues in the body, including pancreatic ductal and acinar tissue ^43^. The use of MSC also avoids some of the ethical issues with the use of EPSCs. However, preclinical studies to date failed to support the notion that MSCs will provide a viable source of mature β-cells and further research is required to establish the therapeutic potential of MSC as β-cell replacement therapy ^24^.

## Preclinical studies-cellular reprogramming as β-cell replacement approach

Recent work, primarily done in mouse models, provided support for the possibility of direct reprogramming of non-β-cell cell types toward insulin-producing and secreting lineage *in-vitro*. Early studies have employed a strategy of forced ectopic expression (typically adenoviral vector-driven) of transcription factors mediating key roles in pancreatic β-cell development (e.g. PDX-1, NGN3 and Insulin). For example, forced expression of PDX-1 and NGN-3 has been shown to promote insulin production and partial conversion toward β-cell lineage in hepatocytes and pancreatic alpha cells ^44,45^. More recently, Zhou and colleagues reported *in-vivo* reprogramming of exocrine acinar cells towards β-cell lineage utilizing an adenoviral vector delivery of three key β-cell transcription factors (NGN3, PDX1 and Mafa) which were directly administered into the exocrine pancreas of mice ^46^. These newly reprogrammed β-cells expressed key markers of β-cell development (e.g. Glut2, GCK and NKX 6.1), displayed ultrastructural characteristics of mature β-cells and reduced hyperglycemia following streptozotocin (STZ) administration to mice ^46^.

Utilizing an alternative approach, Talchai and colleagues recently reported capacity to reprogram cell fate toward β-cell lineage in entero-endocrine gut cells by cell specific deletion of the gene encoding forkhead box 01 (FOXO1) ^47^. Specifically, investigators demonstrated that either fetal or adult ablation of FOXO1 in entero-endocrine NGN3^+^ progenitor gut cell populations promotes reprogramming of gut cells towards β-cell lineage. Importantly, these gut-derived insulin-producing cells were characterized by expression of β-cell maturity factors, glucose-stimulated insulin release *in-vitro* and were capable of reversing hyperglycemia following STZ administration in mice ^47^. This study suggests that genetic repression of original cellular fate transcriptional program also provides an alternative approach to steer cell fate differentiation toward β-cell lineage. Although reprogramming studies provide an intriguing possibility as a potential source of new β-cells, therapeutic potential and long term feasibility of this approach as a β-cell replacement strategy in diabetes remains to be elucidated.

## Conclusion

As should be apparent from this review, the regulation of insulin secretion in response to changing metabolic conditions is complex and dependent on interplay of many signaling systems. Moreover, assuming that said signals are integrated appropriately, the response of the β-cells requires the ability to synthesize and secrete functional insulin molecules. Efforts to date intended to capitalize on current knowledge of islet development and stimulus-secretion coupling of the β-cell are encouraging but as yet of little clinical relevance. With that said, understanding the magnitude of the task ahead is a necessary step towards developing viable cell-replacement therapy for diseases characterized by absent or dysfunctional islets.

## Learning Objectives

- Identify the characteristics of functional, mature β-cells.
- Differentiate the various approaches being explored to generate cellular replacement therapy for Type 1 Diabetes
- Assess the clinical utility of the current efforts to provide β-cell replacement therapy.
